# Physiotherapy and its service in Nepal: implementation and status reported from facility surveys and official registers

**DOI:** 10.1186/s12913-024-10747-0

**Published:** 2024-03-06

**Authors:** Nishchal Ratna Shakya, Nistha Shrestha, Gillian Webb, Hellen Myezwa, Biraj Man Karmacharya, Ann-Katrin Stensdotter

**Affiliations:** 1 Faculty of Medicine and Health Sciences, Department of Neuromedicine and Movement Science, Norwegian University of Science and Technology (NTNU), 7491 Trondheim, Norway; 2 Department of Physiotherapy, Kathmandu University School of Medical Sciences (KUSMS), Dhulikhel, Kavre, Nepal; 3 Epidemiology and Disease control division, Department of health services, Ministry of Health and Population, Government of Nepal, Kathmandu, Nepal; 4 Faculty of Medicine, Dentistry and Health Sciences, The University of Melbourne, Melbourne, Victoria Australia; 5 School of Therapeutic Sciences, University of the Witwatersrand, Johannesburg-Braamfontein, Gauteng South Africa; 6 Department of Public Health, Kathmandu University School of Medical Sciences, Dhulikhel, Kavre, Nepal

**Keywords:** Delivery of health care, NCDs, chronic diseases, health workforce, Health policy, Health services, rehabilitation

## Abstract

**Background:**

Physiotherapy is a growing profession in Nepal. Despite efforts to promote strengthening and development, there are still challenges in providing equitable access and availability to services, particularly in underserved areas. Updated information is needed to address challenges to provide proper planning for resource allocation.

**Objective:**

To assess implementation of physiotherapy services and to explore plans, policies and the general status of physiotherapy in Nepal.

**Method:**

Implementation was assessed with a cross-sectional survey conducted in Province III containing closed-ended questions addressing physiotherapy services, human resources, charging and record-keeping systems, and accessibility. Stratified purposive sampling was used to select eligible facilities from the list of Department of Health Services. Official records were explored through visits to governing institutions and by reviews of registers and reports to obtain data and information on status, plans and policy.

**Results:**

The survey included 25 urban and 4 rural facilities, covering hospitals and rehabilitation centres; both public (37.9%) and non-public (62.1%). Most facilities (79.3%) employed physiotherapists with bachelor’s degrees. Average number of visits were 29.55 physiotherapy outpatients and 14.17 inpatients per day. Patient records were mainly paper based. Most (69%) used the hospital main card, while others (31%) had their own physiotherapy assessment card. Most referrals came from doctors. The most offered services were musculoskeletal, neurological, and paediatric physiotherapy. Daily basis charging was common. A single visit averaged 311 Nepalese rupees ≈ 2.33 US$. Convenience for persons with disabilities was reported as partial by 79% of outpatient departments. Official register data showed 313 master’s and 2003 bachelor’s graduates. Six colleges offered physiotherapy bachelor’s degree, whereof one also offered a master’s program. Government records revealed significant progress in physiotherapy in Nepal.

**Conclusion:**

The study highlights variations in physiotherapy services within a province owing to type, size and location, but also unwarranted variations. Despite the progress, implementation of physiotherapy services in the perspective of official records imply a need of systems for proper planning and monitoring. Physiotherapy provision in underserved areas warrants further attention.

**Supplementary Information:**

The online version contains supplementary material available at 10.1186/s12913-024-10747-0.

## Background

Nepal is facing a significant prevalence of non-communicable diseases, disabilities, physical injuries, health consequences of modern lifestyles and hard manual labour, highlighting the need for urgent attention to the prevention and management of these health issues [1–3]. Physiotherapy, as a non-invasive and non-pharmaceutical health profession, plays a vital role in addressing these health challenges, by working across different settings [4, 5]. However, the profession is relatively new in Nepal and not well understood. To further develop and strengthen its role, knowledge of its status and availability is essential.

Physiotherapists are crucial members of multidisciplinary rehabilitation teams and contribute to promoting healthy lifestyles and addressing musculoskeletal, neurological, cardiovascular, and many other conditions across the lifespan [6, 7]. As the demand for rehabilitation services continues to rise in developing countries, the promotion and availability of physiotherapy services are becoming crucial [8]. Rural and remote areas in lower middle-income countries are often underserved by the rehabilitation workforce [9], leading to limited availability of services. Workforce maldistribution, lack of incentives for therapists, and inadequate skills for providing equitable physiotherapy services exacerbate this situation [10]. Patient referrals to physiotherapists [11], affordability, and availability also impact accessibility [12, 13].

Nepal, with a population of 29.2 million people, is classified as a lower middle-income country [14] where approximately two-thirds of the population resides in urban areas [3]. The country is divided into seven provinces, where the healthcare systems differ between the federal, provincial, and local levels [15]. Health facilities are categorized into primary, secondary, and tertiary levels [16], with significant variations in service provision between urban and rural areas. The challenging topography, poor road conditions, and transportation issues contribute to inequitable access to healthcare outside urban regions [17]. Nepal is also prone to natural disasters such as flooding, landslides, and earthquakes, emphasizing the urgent need to assess the availability of services in the country.

Nepal is committed to accelerating universal health coverage by providing basic health services free of charge and implementing social health protection schemes, particularly for poor and vulnerable populations [16, 18]. While efforts are being made to promote physiotherapy services in public hospitals, the Nepal National Health Policy 2019 has also encouraged private and non-governmental organizations to establish physiotherapy services at the federal, state, and local levels [19]. For instance, USAID Physical Rehabilitation Activity along with Handicap International, in partnership with the Government of Nepal, supports 5 private physical rehabilitation centers and 19 public sector physiotherapy units [20]. Private service provision is considered crucial in the field of physiotherapy [21, 22]. Despite the increasing use of private facilities in Nepal [23], the limited availability of health insurance [16] leads to unaffordable out-of-pocket costs. The provision of sufficient infrastructure in terms of suitable premises, equipment, and documentation systems for patient records, is important for improving healthcare [24]. These issues need urgent attention in the physiotherapy sector in Nepal.

Health profession-related activities are overseen by the Nepal Health Professional Council (NHPC), an autonomous government body responsible for registration and policy making [25]. Registered physiotherapists, with a minimum of bachelor’s degree, are represented by the Nepal Physiotherapy Association (NEPTA) [26, 27]. According to the World Physiotherapy Global report 2022, there is an average of 3.6 physiotherapists per 10,000 people worldwide and for the Asia Western Pacific region, this number is 1.5. In Nepal, the estimated number is only 0.8 [27]. Regrettably, attrition of physiotherapy has become a major issue that impacts the overall profession and its future. Unsatisfactory working conditions and lack of carrier development opportunities leads to migration to high-income countries for employment and education [28].

There have been, however, developments, strategies, and plans aimed at developing and improving physiotherapy and rehabilitation in Nepal, initiated by several stakeholders. The Department of Health Services (DOHS), one of the governing bodies under the Ministry of Health and Population (MOHP), is responsible for addressing the aforementioned issues [29]. Its objectives include providing preventive, promotive, and curative health services, providing technical advice for health policies. It is also responsible for developing and expanding health institutions, determining manpower requirements, encouraging participation from the private sector and international institutions. Additionally, coordinating and controlling the quality of health services, and maintaining data and information on health services [29].

Despite these efforts, Nepal still faces challenges in implementation resulting in huge gaps between plans and actions in all sectors [30]. It is thus essential to gain updated information about the number and geographic distribution of the workforce and physiotherapy availability to enable planning of current and future resource allocation. To address this issue, a province-level survey in combination with exploration of official records was undertaken. The survey aimed to assess implementation of physiotherapy services in different health facilities in a selected province. The assessment was focused on objective measures that could be verified such as type of services, human resources, charging and documentation systems, and accessibility. Official records were explored for policies and plans, and the general status of physiotherapy with regard to development in workforce, education, facility and resource allocation, law and regulations. The outcome was expected to reveal the current status of physiotherapy services in the perspective of governing policies and plans.

## Methods

### Design and setting

Several of the authors own special insight into the Nepal health service, academic and governing systems. The first author, NRS, is a senior physiotherapy faculty member and clinician at KUSMS, and former president of NEPTA. NS is a government employed physiotherapist working under DOHS. BMK is the director of the Public Health program at KUSMS. GW has worked with development of physiotherapy in South-East Asia for more than 20 years.

To assess implementation of physiotherapy service, a cross-sectional survey was conducted at province-level. The Bagmati region, Province III was selected as a fair representative for Nepal having the greatest proportion of registered physiotherapists (71%) [31] and being the second most populated area (20.84%), encompassing diverse ethnicities, all geographical zones, having complete administrative divisions and including the capital city of Kathmandu [3]. The districts and health facilities were selected using a stratified purposive sampling method [32] aimed at maximum variation [33, 34] between types and locations of facilities that filled the inclusion criteria. Out of 13 districts in the province, six (Kathmandu, Bhaktapur, Lalitpur, Kavrepalanchowk, Makwanpur, Dolakha) were selected to secure a mix of urban-rural settings with geographical and cultural diversities. While the on-site survey at health facilities was limited to Province III, the official reports and registers provided information at national level.

### Data acquisition

#### Survey data

**Fig. 1:**
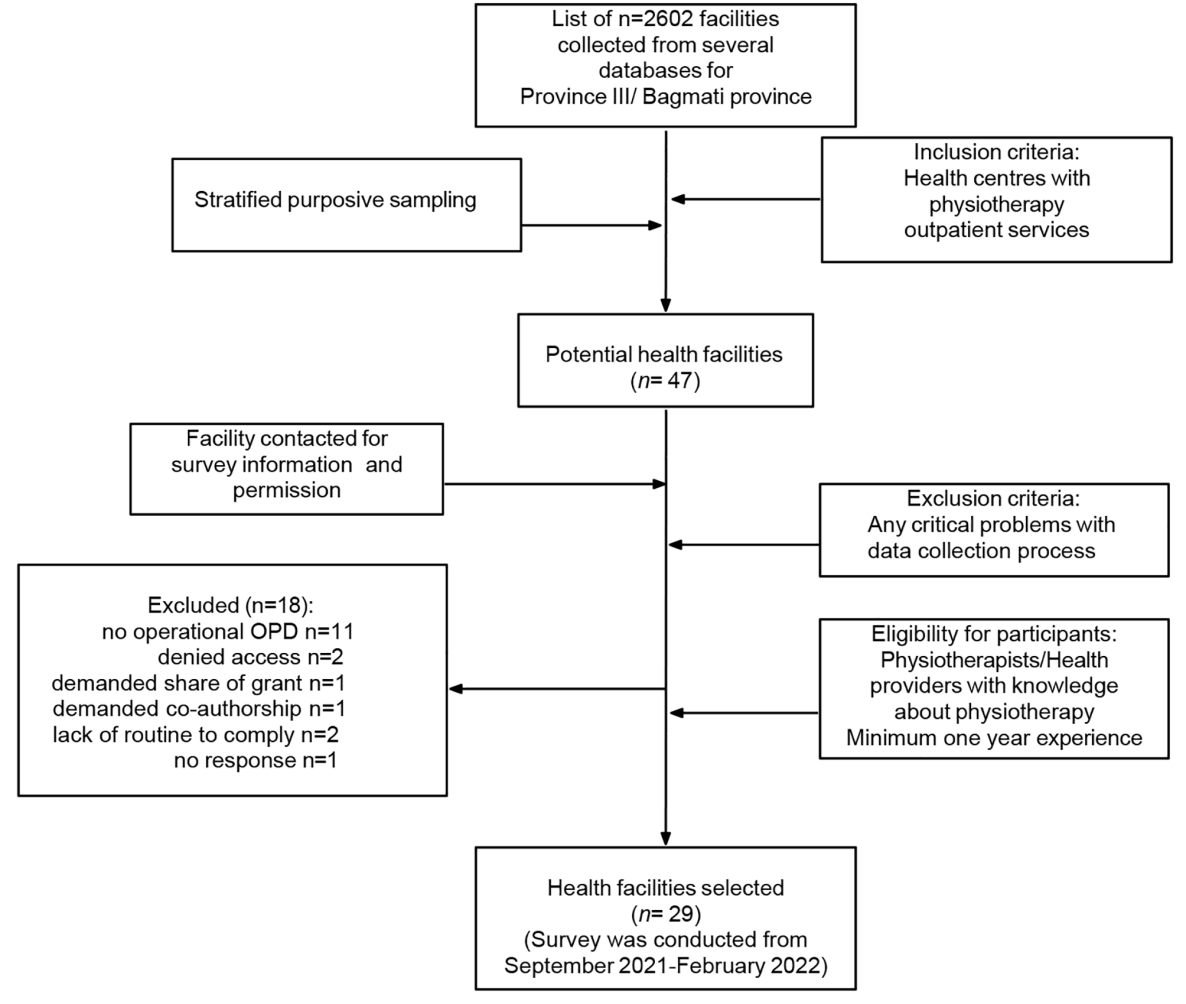
Process of selecting health facilities for the survey (OPD: Outpatient department)

A list of facilities was collated from the DOHS website [35], publicly available online directory. The list included 1450 non-public and 1139 public health facilities and 13 medical colleges in Province III. Facilities were extracted for the six districts selected for geographical location. Availability of physiotherapy services were verified based on information from websites and by contacting officers in charge for confirmation about service provision and alignment with the eligibility criteria. The criteria included provision of outpatient physiotherapy, at least one physiotherapist on duty and that the health professional in charge had minimum one year of working experience in the facility to be able to give appropriate information. Listed facilities were then categorised into tertiary, secondary, medical college centres, rehabilitation and physiotherapy centres with a selection from a mix of metropolitan, urban-suburban and rural districts. Of the 47 potential centres shortlisted, 29 facilities were included in the survey (Fig. 1). The survey consisted of closed-ended questions divided into two sections: (1) information on the health facility in general, and (2) physiotherapy-related data divided into three categories: (a) physiotherapy services and human resources, (b) charging and physiotherapy record-keeping system, and (c) accessibility (find complete survey in the additional file). The survey questions were pilot tested at five different centres and adjusted based on feedback for content and clarity. Physiotherapists or other primary contacts were identified for field visits and information about the survey was provided through email and phone calls. A reminder was sent prior to the visit. Data was collected on site by a research assistant trained and supervised closely by the first author (NRS) to ensure high-quality data collection. Survey data (Table 1) were recorded using a tablet in the Kobo data toolkit [36, 37].

**Table 1:** Facilities providing physiotherapy services

Services and human resources	n (%)
**Type of physiotherapy services**	
Outpatient services only	8 (27.6)
Both outpatient and inpatient services	21 (72.4)
Type of physiotherapy intervention*	
Endurance training, resistance training, flexibility, balance, gait training and motor re-learning	29 (100)
Electrotherapy, manipulation and mobilisation, supervised training in clinics, consultation services	28 (96.6)
Home programs	22 (75.9)
Massage therapy	2 (6.9)
Other physiotherapy services: sports physiotherapy, functional training, kinesiology taping, hydrotherapy, school health program, NDT (neuro developmental treatment), sensory integration, oromotor training, functional training, dry needling and acupuncture.	5 (17.2)
Specialist with Master’s degree (NHPC categories)*	*n* (%) denotes number of facilities employing specialist
Musculoskeletal and sports	15 (51.7)
Neurological and psychosomatic disorders	8 (27.6)
Paediatrics	5 (17.2)
Cardio-pulmonary science	2 (6.9)
Health promotion and rehabilitation	0
**Specialist other than NHPC category** (CBR-3, Rehabilitation officer-2, sports-1)	6 (20.7)

*Denotes multiple choice options, therefore the percentage does not total to 100

NHPC: Nepal Health Professional Council

CBR: Community based rehabilitation

#### Register and report data

Key public stakeholders and national professional organisations were selected to obtain relevant data on the status of physiotherapy in terms of the workforce, education, and policies. Key contacts were identified, and permission was obtained to acquire the available data. Statistics on the registered physiotherapists were obtained from the NHPC and NEPTA. Data from governing institutions had to be retrieved through personal visits of the first author (NRS). This included MOHP, the Medical Education Commission (MEC), and the Leprosy Control & Disability Management Section (LCDMS) under DOHS. National and international data from reports, journals, documents, websites, and formal and informal records regarding the workforce and recent developments were also included.

### Ethics and permissions

Ethical approval was obtained from the Nepal Health Research Council-Ethical Review Board (NHRC-ERB Protocol No. 455/19), the Institutional Review Committee of Kathmandu University School of Medical Sciences / Dhulikhel hospital (IRC-KUSMS 104/18), and the Norwegian Centre for Research Data (NSD 383963). Permission was obtained from all the facilities included in the survey. Written informed consent was obtained from each participant prior to enrolment. Survey responses were anonymous, i.e., without identification or keys that could connect the responses to the consent form. The survey did not contain personal identifiable data that could connect the participant to a district or health centre. The data was securely stored on the university server.

### Data analysis

A descriptive frequency analysis of the survey data was conducted using SPSS 29. Microsoft Excel was used to compilate and organize information from registers and reports [38].

## Results

### Survey data

Of the 47 facilities contacted, 38 were in urban and 9 were in rural areas. Two rural districts, Sindhuli and Dhading with populations of 300,117 and 322,751 respectively, did not have operational physiotherapy services during the data collection period. Two other rural districts, Rasuwa and Ramechhap, with populations of 45,554 and 170,620 respectively [3], had only one centre each; one centre did not respond and the other was excluded due to unsafe road conditions. The final sample included 29 health facilities, 25 in urban areas (12 in metropolitan cities and 13 in sub-metropolitan areas of Kathmadu and Lalitpur) and 4 in rural areas of Dolakha. The facilities included hospitals (*n* = 21) and rehabilitation centres (*n* = 8) which were a mixture of public (37.9%) and non-public (62.1%) settings including private and non-governmental clinics.

The distribution of the workforce varied regarding the type, size, and location of the facility, with one facility having 18 physiotherapists and most having 1–4 physiotherapists. Most facilities (*n* = 21) employed at least one physiotherapist with a master’s degree where the specialties differed centre wise (Table 1). Seven facilities had only a bachelor’s degree-level physiotherapist, one lacked a physiotherapist with a university degree having only one staff member with 3–18 months of vocational training. The majority of the workforce had a bachelor’s degree (*n* = 67), followed by a master’s degree (*n* = 50).

**Fig. 2:**
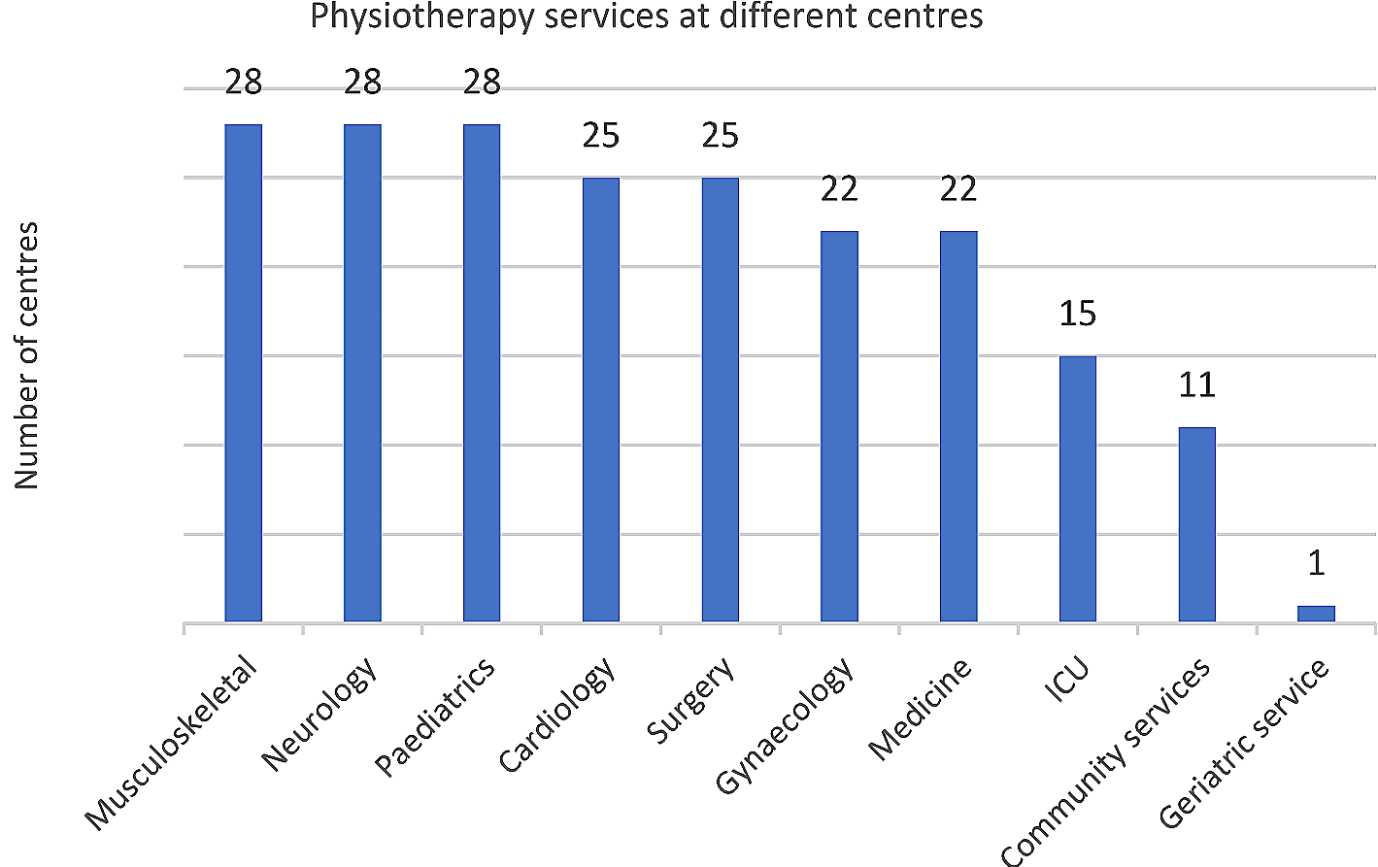
Physiotherapy services availability (ICU: intensive care unit)

The types of physiotherapy services provided varied between facilities. Hospitals with outpatient and inpatient physiotherapy services offered greater variety and more specialities compared to smaller rehabilitation centers (Fig. 2). The most commonly offered physiotherapy services in the wards in larger facilities were musculoskeletal, neurological, and pediatric physiotherapy, with many facilities also offering physiotherapy services for other medical conditions. One centre provided exclusively geriatric services, while another centre, though having a physiotherapy outpatient department (OPD), primarily focussed on distributing orthotics and prosthetics due to resource constraints.

**Fig. 3:**
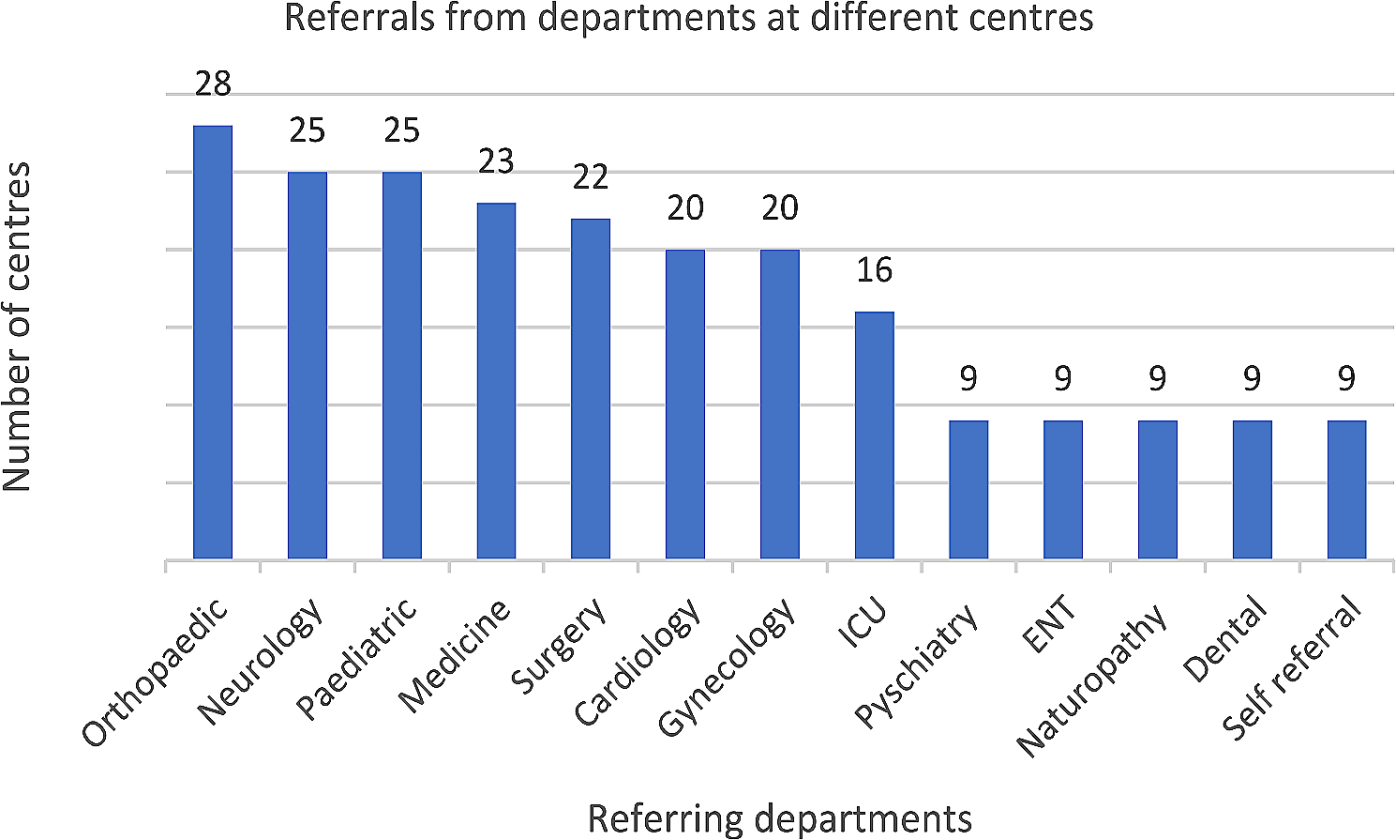
Referrals to physiotherapy. ICU: Intensive care unit; ENT: Ear, nose and throat

The average number of physiotherapy outpatients per day was 29.55 (range 3–80) and inpatients was 14.17 (range 2–30), which also varied depending on type, size, and location of the facility in the selected six districts of the province. The referrals came mainly from orthopaedic, neurology, and paediatrics departments. Some patients were self-referrals coming directly to outpatient clinics (Fig. 3).

**Fig. 4:**
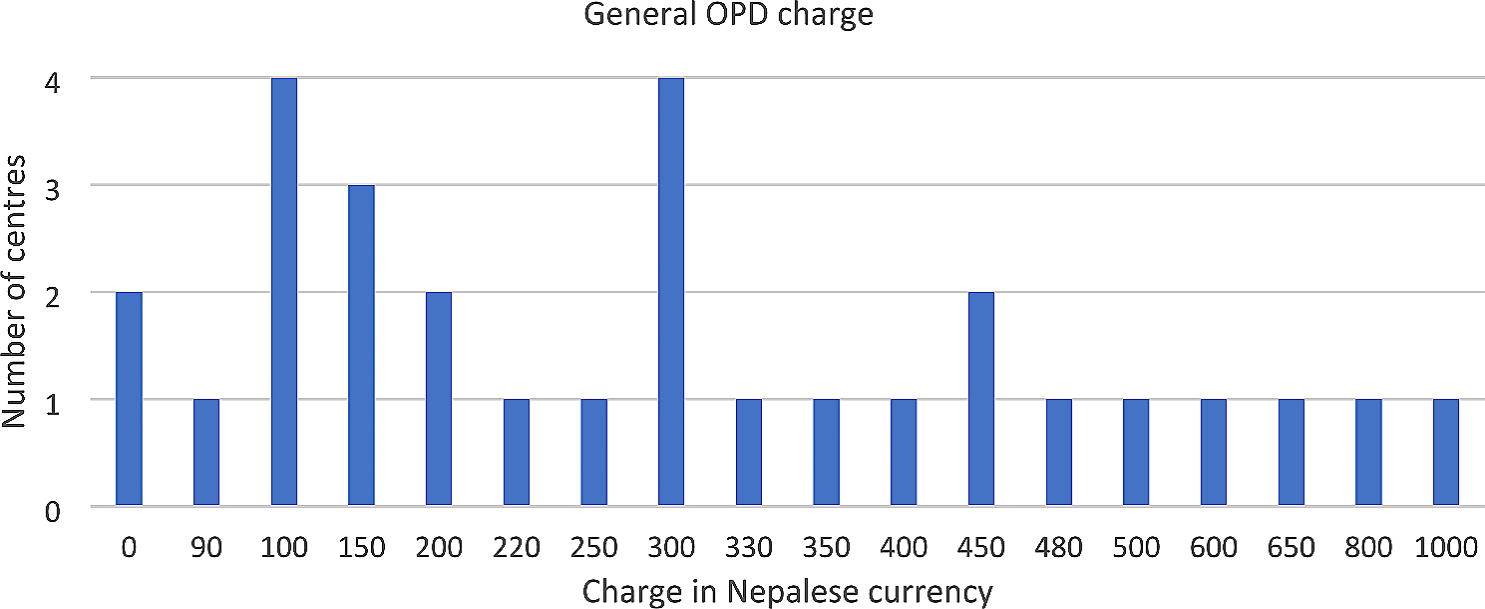
Range of charging at OPD (outpatient department) for physiotherapy services for first visit

The average charge for a first or single visit at a physiotherapy OPD was 311 Nepalese rupees (Npr) ≈ 2.33 US$, but varied greatly among the included OPDs, ranging between 90 and 1000 Npr for a single visit, while two centres reported providing free treatments (Fig. 4). Charges for follow-up treatments ranged from less than 100 to more than 500 Npr. Most centres (*n* = 27, 93.1%) reported charging on a daily basis. Other charging systems included package-based, modality-based, or treatment-based charging.

Patient records were mainly paper based, with most OPDs (*n* = 20, 69%) using the hospital main card for patient case documentation, while others (*n* = 9, 31%) had their own physiotherapy assessment card.

Accessibility for persons with disabilities was reported as partial by 79% of the OPDs, foremost with regard to wheelchairs, providing a ramp, elevator, or stretchers. Two centres reported not having any disability-friendly services, while only one centre reported having a disability-friendly toilet.

### Register and report data

#### Physiotherapy workforce details

The NHPC database provided information on the registrations and numbers of physiotherapy professionals categorised under different levels. A master of physiotherapy was registered under five different specialisations (Table 1). Year-wise estimated records of the number of physiotherapists in Nepal from 2018 to 2023 were obtained from a workforce survey [28], the World Health Organisation (WHO) [39], World Physiotherapy [26], DOHS [40], and NEPTA [27]. According to NEPTA, the estimated number of physiotherapists in Nepal has increased 63% from 1587 (WHO, 31) in 2018 to approximately 2500 in 2023, while DOHS reported only 1200 for 2022. The number of registered physiotherapists in the Nepal Health Workforce Projection Report 2022–2032 [41] published in August 2023 was 2022 physiotherapists. Updated verbal information from the NHPC record (December 2023) revealed 313 master’s and 2003 bachelor’s graduates (total *n* = 2316).

#### Practice and academia initiatives

Physiotherapy practise in Nepal began in 1972, and in 2003 a certificate-level program was launched [42]. This program was upgraded to a bachelor’s level program in 2010 by Kathmandu University School of Medical Sciences [43]. Two colleges under Kathmandu and Pokhara University offer full-time bachelor programs [44], with four more colleges recently added to the list providing a total of 150 seats under the affiliation of Kathmandu University [45]. In the fall of 2022, the first master’s program in physiotherapy was accredited at Kathmandu University [46], which currently has an annual intake of five master’s graduates specialising in musculoskeletal physiotherapy.

#### Strategy, plans, and policies related to physiotherapy

Government databases, journals, and reports revealed significant work in progress. After the earthquake in 2015, the National Health Sector Strategy III, 2015–2020, referred to physiotherapy as part of Basic Health Services. The MOHP has included standards for physiotherapy under the minimum service standards for secondary and tertiary hospitals. The National Strategy for Reaching the Unreached 2016–2030 includes the establishment of physiotherapy units in hospitals [31, 47]. According to the DOHS annual report 2020/21 [40], physiotherapy clinical protocols and standard operating procedures were developed. The report highlighted the strengthening of existing physiotherapy units and promotion of the establishment of new units in government hospitals [40]. However, physiotherapy services are lacking in secondary hospitals and government primary care facilities [31].

## Discussion

This study provides the first comprehensive overview of physiotherapy service provision in Nepal. Inconsistencies in baseline data on the number of registered physiotherapists call for the need of systematic records. The survey revealed significant geographical imbalances and varying levels of availability and distribution of physiotherapy within Province III. Our findings have implications for decisions on resource allocation and contribute to facilitating more logical physiotherapy allocation and may serve to guide implementation in other provinces.

Our study supports the notion that physiotherapy services are primarily concentrated in urban health settings, as reported in previous study by Nepal et al. (2022) and for other countries [10, 22, 28]. This finding corroborates a previous report in which 71% of physiotherapists in Nepal were found to be concentrated in Province III [31]. One previous study suggested that the distribution of physiotherapists can be related to population density and monetary resources, as well as other potential influencing factors such as the availability of public and private services, or professional opportunities in cities [48]. However, the limited availability or lack of physiotherapy services in rural districts despite significant population density, as reported in our study, could be due lack of prioritization or awareness about the service, or lack of attracting working conditions [49].

Our results revealed a low frequency of physiotherapists particularly in cardio-pulmonary and paediatrics specialisations, although the availability of and referral for such physiotherapy services at the surveyed facilities were significant. This finding is consistent with the World Physiotherapy interview report that highlighted the key challenges faced by Nepali physiotherapists in recognizing specialty-based physiotherapy practices [49]. This may also be reflected in a Canadian study that indicated physiotherapists generally assume a generalist role, providing different clinical services to their patients [50]. Our study also revealed a physiotherapy unit operated by a non-qualified physiotherapist, indicating the need for proper implementation of hospital service monitoring and strengthening by concerned governing bodies such as the Bagmati Province Health Directorate [51], DOHS [52] and health sections at local governments. Such practice is also in defiance of World Physiotherapy and NHPC regulations where authorization as a registered physiotherapist requires a minimum bachelor’s degree in physiotherapy for clinical practice [26].

Our study showed that access to physiotherapy heavily relies on doctor referrals from various departments. However, studies have suggested that direct access to physiotherapy via self-referral [53, 54] could contribute to increased availability and a reduced healthcare burden on doctors without significant risk to patients [55, 56]. The present study also revealed inconsistent and varied charging systems in most of the physiotherapy facilities, raising concerns about the quality of services and out of pocket costs. Unpredictability in charges is a serious concern particularly for the 15.1% of Nepalese population reported below the poverty line with a daily purchasing capacity at < $1.90 [57, 58]. Gross salary ranges between 15,994 and 60,346 Npr [59], where one income often sustains a large extended family [60]. Unemployment was in 2022 estimated to 11.1% [61]. Predictable and fair charging systems need to be reconsidered by stakeholders exemplified by practices in other countries [62, 63]. Our study also revealed that most Nepali physiotherapy facilities use hospital OPD cards for documentation. Only a few had their own physiotherapy evaluation forms, indicating a lack of systematic documentation. Our findings on challenges associated with physical accessibility and disability-friendly design were consistent with findings of the Nepal health facility survey 2021, which showed that most health facilities lacked accessible doors, entrances, corridors, ramps, and toilet conveniences [16].

During our data collection, the human resource division at the MOPH struggled to provide information about physiotherapy public service availability and the number of physiotherapists employed. This is also reflected in the Nepal Health Fact Sheet 2023 [64] derived from the Health Information Management System (HMIS) [65]. The health human resource and service coverage section of the fact sheet does not include physiotherapy specific data. The discrepancies in the reports collected in our study on the numbers of registered physiotherapists, implicates the need for a systematic approach and authority to gather reliable baseline information. Despite recent initiatives and policy reforms, we still emphasize the need of proper documentation in the national databases on registered physiotherapists and monitoring of physiotherapy services. It is important that policy and decision makers should not further delay prioritising and promoting physiotherapy services as part of public health services. This is particularly important to meet the requirements and needs in remote and underserved areas [66] where private practices cannot be sustained partly due to affordability issues [56]. In compliance with our results and others [48], physiotherapy academic schools and professional organisation in Nepal need to advance education for skills to meet demands in areas with identified short supply. Financial support would be required for such initiatives. To further increase the availability of physiotherapy within the unique Nepalese context, telerehabilitation could be an option as a supplement for serving rural and remote areas [9].

### Strengths and limitations of the study

This is the first multi-site approach in various healthcare settings to assess particularly the availability of physiotherapy services in Nepal. This is also the first study to explore the updated data from registries and different official reports related to physiotherapy. A limitation was that the investigated sites were not evenly distributed, which made it difficult to draw conclusive distinctions between rural and urban settings. Still, the findings hold internal validity serving as evidence to inform policy and planning in Province III. We also believe that the results can contribute to direct attention toward challenges in implementation of physiotherapy services in Nepal, although external validity may be compromised due to significantly lower density of physiotherapy service in other provinces and differences in provincial health systems organization.

## Conclusion

Overall, the study highlights some significant variations in physiotherapy services among districts within a province owing to type, size and location, but also unwarranted variations of great differences in charging rates and means of keeping patient records. In general, investigated facilities appear properly staffed with authorized physiotherapist with university degrees. On the downside was unsatisfactory accessibility for persons with disabilities. Official records show an expansion in human resources, improvements in the educational systems with plans and policies pointing in a positive direction. The resulting implementation of physiotherapy services in the perspective of official reports and registries imply however a necessity of improved steering documents and systems for proper planning and monitoring of the development of physiotherapy services. Physiotherapy provision, particularly in underserved areas, still lags behind warranting further attention.

### Electronic supplementary material

Below is the link to the electronic supplementary material.

Supplementary Material 1

## Data Availability

Data is provided within the article and supplementary information file.

## Declarations

### Authors’ information

The authors involved have varying levels of professional qualifications. NRS is a senior physiotherapy academician and clinician at KUSMS, former president of NEPTA and a Ph.D. scholar at NTNU. NS is a government employee working under DOHS, Nepal. All the authors are actively involved in national and global collaboration. All the authors have a physiotherapy background, while BMK is an expert in public health and the Nepalese health system. All the authors have significant engagement in global health especially in health promotion, non-communicable diseases, disability, physiotherapy, and rehabilitation.

## Abbreviations

CBR: Community-Based Rehabilitation
DOHS: Department of Health Services
MOHP: Ministry of Health and Population
MEC: Medical Education Commission
NEPTA: Nepal Physiotherapy Association
NHPC: Nepal Health Professional Council
OPD: Outpatient department
