# Supplementary material for: Physiotherapy and its service in Nepal: implementation and status reported from facility surveys and official registers
Source: BMC Health Serv Res. 2024 Mar 6;24:295. doi: 10.1186/s12913-024-10747-0 (PMC10918904; doi:10.1186/s12913-024-10747-0)
Supplement: Supplementary file 1 — Supplementary Material 1 [file 12913_2024_10747_MOESM1_ESM.docx]

***I. Questions - facility***

**Facility Identification**

Facility code:

Locations:

Do you wish to provide consent for this research? Yes/No

1. Name and type of municipality

- Urban Municipality, specify the name
- Rural Municipality, specify the name

1. Facility type

- Hospital
- Rehabilitation center
- INGO
- NGO
- Other, mention if other:

If hospital,

- Government
- Private
- Polyclinic
- Community
- Public University Hospital
- Private University Hospital
- Tertiary healthcare center
- Secondary healthcare center
- Primary healthcare center
- Ayurvedic Hospital
- Naturopathy Hospital

**General facility detail**

1. Year of establishment (AD):
2. Total number of beds:
3. Average number of outpatients per day:
4. Average number of inpatients per day:
5. Department providing services

- Orthopaedic
- Neuro
- Cardio
- Paediatrics
- Gynecology
- Medicine
- Surgery
- Dental
- ENT
- ICU
- Community
- Physiotherapy
- Occupational Therapy
- Orthotic and Prosthetic
- Speech Therapy
- Other rehabilitation services, mention if other:

**Physiotherapy related data**

1. Physiotherapy services

- Outpatient services only
- Both outpatient and inpatient services
- Other

1. Physiotherapy services available

- Musculoskeletal
- Neuro
- Cardio
- Pediatric
- Women's Health
- Medicine
- Surgery
- ICU
- Community
- Other services

1. Average number of physiotherapy outpatients per day?
2. Average number of physiotherapy inpatients per day?
3. Types of services offered

- Electrotherapy
- Endurance Training
- Resistance training
- Flexibility
- Balance
- Manipulation/Mobilization
- Gait Training
- Motor re-learning
- Supervised training in clinic
- Home Program
- Rehabilitation service
- Consultation
- Massage
- Others

1. Referring departments to physiotherapy

- Orthopedic
- Neurology
- Cardiology
- ICU
- Pediatrics
- Medicine
- Surgery
- Gynecology
- Others

1. Physiotherapists or Physiotherapy assistant available

- MPT
- BPT
- CPT
- Others

*Specify the available number of each human resources.*

1. MPT – Specialist

- Musculoskeletal-Physiotherapist
- Neuro-physiotherapist
- Cardio-physiotherapist
- Pediatric Physiotherapist
- Other, specify:

**Charging system**

1. General OPD charge on first day:
2. What are the different charging systems?

- Daily basis charging system
- Different follow up charging system
- Package based charging system
- Modality/Treatment based system
- Other, specify:

If different follow up charging system:

- <100
- 100-199
- 200-299
- 300-399
- 400-499
- 500 and above

**Physiotherapy record-keeping system**

1. Who maintains the patient registration?

- Hospital/clinic/facility
- Physiotherapy department
- Both
- Others, specify:

1. How is patient data/registration recorded?

- Electronic recording
- Paper-based recording
- Both
- Others, specify:

1. How is patient case documentation done?

- Patient's hospital/facility OPD card
- Physiotherapy OPD card
- Separate physiotherapy assessment form
- Others, specify:

**Disability friendly center**

1. Availability of infrastructure for OPD access for persons with disabilities

- Wheelchair
- Ramps
- Elevator
- Stretchers
- None
- Others

1. Support for access to OPD for persons with disabilities

- Assistance by helpers/ward boys
- Self-assistance-self/family/friends
- Both

1. Which floor is the physiotherapy OPD?

- Basement
- Ground floor
- First floor
- Second floor
- Third floor
- Fourth floor
- Fifth floor
- Sixth floor
- Seventh floor

1. Overall, how disable friendly is your hospital/facility?

- Not at all
- Partially
- Very disable friendly.
